# Pediatric Acetabular Osteomyelitis Treated With Hip Arthroscopy

**DOI:** 10.5435/JAAOSGlobal-D-21-00011

**Published:** 2021-05-04

**Authors:** Lisa J. Lovse, Stephanie A. Coupal, Andrew D. W. Tice, Nicole Le Saux, Sasha P. Carsen

**Affiliations:** From the Division of Orthopaedic Surgery, Western University, London, Ontario, Canada (Dr. Lovse); the Division of Orthopaedic Surgery, University of Ottawa, Ottawa, Ontario, Canada (Dr. Coupal); the Division of Orthopaedic Surgery, CHEO, Ottawa, Ontario, Canada (Dr. Tice, Dr. Carsen); and the Division of Infectious Diseases, CHEO, Ottawa, Ontario, Canada (Dr. Le Saux).

## Abstract

Osteomyelitis of the acetabulum is a rare condition accounting for only 12% of pelvic osteomyelitis cases. This report describes a previously healthy 10-year-old girl with subacute acetabular osteomyelitis and subsequent development of secondary septic arthritis of the hip. The patient presented with 3 weeks of groin pain, elevated erythrocyte sedimentation rate and C-reactive protein, synovial thickening of the hip on ultrasonography and diffuse signal uptake in the acetabulum on magnetic resonance imaging. Despite antibiotic therapy, her symptoms worsened clinically, and repeat Magnetic resonance imaging images showed worsening of the osteomyelitis with likely extension through the acetabulum and into the joint. A hip aspirate was positive for *Fusobacterium*, an atypical anaerobe. Hip arthroscopy, with identification of the site of extrusion and then extensive débridement and irrigation, was successful in helping to control and ultimately eradicate the infection. The patient regained normal hip function and returned to full activities. This case demonstrates how hip arthroscopy can serve as an important surgical treatment modality for acetabular osteomyelitis with intraarticular extension in addition to septic arthritis of the hip.

Acute pediatric osteomyelitis is relatively uncommon, occurring in approximately 8 of 100,000 of children per year.^1^ Pelvic osteomyelitis is a small subset (3% to 12%) of these cases, and diagnosis can often be delayed until the infection spreads to the adjacent hip and more apparent symptoms are recognized.^2^ Pelvic osteomyelitis is essential to recognize as early as possible because it can often be treated effectively with systemic antibiotics alone when diagnosed and treated in a timely fashion. However, the development of adjacent septic arthritis of the hip requires more urgent surgical intervention.^3^ The sequelae of septic arthritis of the hip, particularly when identified late or missed, can be severe, including destruction of the proximal femoral physis, osteonecrosis of the femoral head, progressive limb length discrepancy, ankylosis of the hip joint, or instability of the hip.^4^ Here, we present the case of a 10-year-old girl with subacute acetabular osteomyelitis and secondary septic arthritis of the hip, successfully treated with arthroscopic débridement and lavage along with systemic antibiotic therapy.

## Case Report

**Figure 1:**
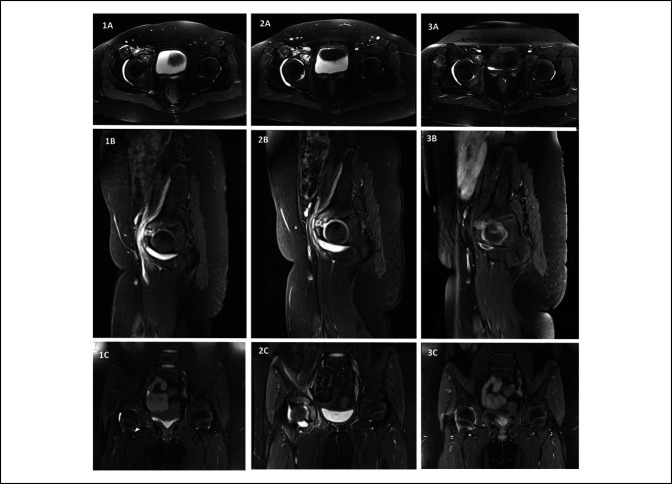
Radiograph showing the T2-weighted pelvic MRI images (**A** axial, **B** sagittal, and **C** coronal) of the patient (1) at the time of admission, (2) immediately preoperatively, and (3) 2 months postoperatively.

A healthy 10-year-old girl presented to the pediatric emergency department with a 3-week history of right groin pain. Initial bloodwork showed an elevated white blood cell count (WBC) of 11.3, an elevated erythrocyte sedimentation rate of 37, and an elevated C-reactive protein of 149.7. A pelvic radiograph was normal. Ultrasonography of the right hip demonstrated mild synovial thickening and a small hip effusion. Because of a concern for pelvic osteomyelitis, she was admitted to hospital under the care of the pediatric medicine and infectious disease teams. She was treated with empiric IV antibiotic therapy using a broad-spectrum first-generation cephalosporin (cefazolin) based on regional infectious epidemiology. A magnetic resonance imaging (MRI) was done on postadmission day 1 and was consistent with anterior acetabulum osteomyelitis (Figure 1) and demonstrated increased hip fluid suggestive of a reactive effusion with synovitis. An image-guided joint aspiration was done the same day. The aspirate showed a cell count of 14,889, consisting of 92% polymorphonuclear cells, and Gram stain with no organisms but many polymorphonuclear leukocytes. Bacterial growth of *Fusobacterium*, an atypical anaerobe, was reported 6 days later. At that time, metronidazole was added to her antibiotic regimen.

Despite antibiotic therapy, minimal improvement was observed in the patient's symptoms, and she remained unable to put notable load through her right hip. An MRI was repeated 9 days after admission and showed progression of the right acetabular osteomyelitis with two areas suggestive of necrosis near the junction of the pubis and ilium. There was an increase in the size of the joint effusion and demonstration of breach in the cortex and cartilage with intraarticular progression. The orthopaedic surgery service was then consulted urgently.

**Figure 2:**
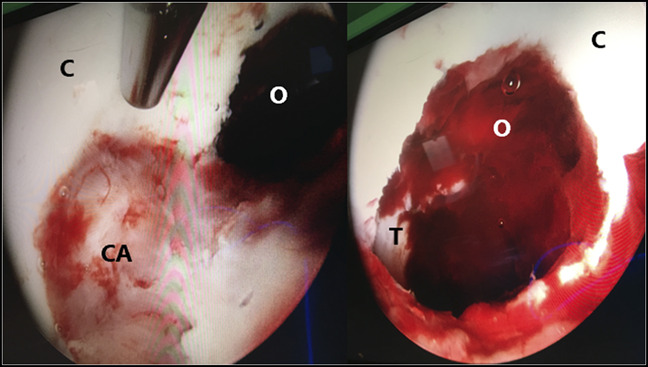
Intraoperative arthroscopic photographs showing the acetabular cartilage (C), osteomyelitis lesion (O), central acetabulum (CA), and triradiate cartilage (T). Of note, the lesion is of a considerable size, measuring 8 × 10 mm after final débridement, and extends past the cartilaginous joint surface and down to the level of the triradiate cartilage.

The patient was booked for urgent irrigation and débridement of the right hip. An arthroscopic approach was used to allow for visualization of the extent of cortical breach inside the acetabulum. The patient was placed supine on a hip distractor table extension, and a fluoroscopically assisted joint aspirate was done. Subsequently, standard arthroscopic anterolateral, posterolateral, and mid-anterior portals to the hip were developed. Within the hip joint, notable debris and turbid fluid was observed, consistent with a septic joint. The cartilage of the femoral head appeared normal in condition. The anterior portion of the acetabular cartilage was extremely friable, and an avascular lesion measuring 5 × 5 mm was apparent (Figure 2A). The damaged cartilage was debrided to reveal a cavern of necrotic bone behind it (Figure 2B). A sample of the cartilage and necrotic bone were sent for microbiology and pathology. An extensive débridement and osteoplasty were completed with arthroscopic shavers, down to normal bone. The patient was brought back to the operating room 48 hours later for a repeat arthroscopic irrigation and débridement based on a clinical decision made by the combined multidisciplinary treating services, institutional norms, and concerns regarding the atypical bacterial growth. All further necrotic and purulent material was removed. The residual acetabular defect measured approximately 8 × 10 mm.

The patient's symptoms improved dramatically after surgical intervention. Her C-reactive protein was 33.6 on postoperative day (POD) 2, 25.1 on POD 4, 9.4 on POD 7, and within normal range 2 weeks after surgery. Pathology results were consistent with acute-on-chronic osteomyelitis. Given the patient's subacute presentation, she was treated with metronidazole for a total of 3 months with transition to oral antibiotics at the 4-week mark. MRI at 2 months postoperatively showed notable interval improvement, with ingrowth into the defect in the acetabulum and fibrocartilage in the area of cartilage deficit. At clinical follow-up 2 years postinfection, the patient was asymptomatic, with a normal gait, and a full return to sport. Informed consent was obtained from the patient's parent for publishing the details of this case and associated images.

## Discussion

Pediatric pelvic osteomyelitis is relatively uncommon; acetabular osteomyelitis occurs even less so. The relatively low incidence can make this condition difficult to diagnosis early, and clinical signs can be mistaken for other more common pediatric conditions, such as appendicitis.^5^ However, a diagnosis of osteomyelitis should be on the differential in patients who present with deep pelvic pain and fever and/or are systemically unwell.^4^ Patients may demonstrate an inability to weight bear on the affected lower extremity, pain to range of motion of the hip, positive Faber test, or pain with compression and distraction of iliac wings.^6^ Because the pelvic bones are deep with minimal movement, the usual signs of long bone osteomyelitis such as tenderness to palpation and limitation of motion are often lacking.^6^ Bloodwork should be drawn to aid in diagnosis and establish a baseline. Blood cultures should also be sent because most pediatric osteomyelitis infections occur secondary to a hematogenous source and positive blood cultures may help with early targeted antibiotic therapy. Radiographs of the pelvis often do not show bony changes in the acute period, although more chronic or acute-on-chronic cases may present with radiographic findings. MRI is the benchmark for diagnosis, with 97% sensitivity and 92% specificity in diagnosing acute musculoskeletal infection.^7^ Early recognition of osteomyelitis and initiation of proper treatment is essential to obtain optimal outcomes and avoid potential severe sequelae such as hip joint necrosis or disturbance of the triradiate cartilage and subsequent abnormal acetabular growth.

Assessing for intraarticular extension and secondary septic arthritis is also very important in the management of pelvic osteomyelitis. The incidence of joint involvement with acetabular osteomyelitis in pediatric patients has been reported to be as high as 66%.^1^ Although ultrasonography is the most sensitive tool for detecting an effusion in the hip, MRI is more specific and may show signal intensity alterations of the bone marrow and contrast enhancement of the adjacent soft tissue.^4^ If a hip effusion is present and there is concern for septic arthritis, a hip aspirate should be done.

The foundation of osteomyelitis treatment is IV antibiotic therapy,^3^ and therefore, a multidisciplinary approach to management is required.^7^ *Staphylococcus aureus* is responsible for 90% of osteomyelitis and septic arthritis cases in infants and children,^4^ but there is regional variation, and MRSA can be a notable risk, especially in endemic areas. Historically, children were treated with intravenous antibiotic therapy for several weeks, but an earlier transition to oral therapy has recently been advocated in patients with uncomplicated cases who respond clinically to IV antibiotics.^8^ Symptomatic treatment can also involve nonsteroidal anti-inflammatory medications.^3^

Surgical indications in the treatment of osteomyelitis include irrigation and débridement of subperiosteal collections or adjacent abscesses, failure to respond to antibiotics, or to obtain tissue samples to help guide selection of antibiotic therapy.^3^ Surgery is also indicated in cases of articular extension to reduce the risk of severe complications such as permanent cartilage damage.^5^ Procedural options for addressing septic arthritis of the hip include aspiration, arthroscopy, and open arthrotomy,^7^ with open arthrotomy still the mainstay and benchmark for treatment in most centers.

Acetabular osteomyelitis and associated septic arthritis have traditionally been treated with open surgical débridement.^5,10^ However, arthroscopic lavage of the hip has been described for treatment of septic arthritis of the hip for both children and adults. There is no previous report of the use of arthroscopic techniques to treat acetabular osteomyelitis. In our case, an arthroscopic approach was chosen because it was felt it would provide the best visualization of the articular defect and enable adequate débridement while preserving healthy cartilage. In choosing the most appropriate surgical approach, it is crucial to determine the precise anatomic location of the infection on perioperative imaging. In addition, it should be recognized that hip arthroscopy can be technically challenging. Importantly, the surgeon is experienced and comfortable with the technique and associated equipment before attempting its use in acetabular osteomyelitis.

## Conclusion

We present a unique case of acetabular osteomyelitis with secondary septic arthritis, treated successfully with hip arthroscopy and antibiotic therapy. This surgical technique should be considered for the experienced hip arthroscopist for management of this relatively rare condition because it provides an excellent view and access to the joint and joint surfaces and allows for excellent débridement of pathological tissues and irrigation of the joint.

## References

[1] Riise ØR Kirkhus E Handeland KS: Childhood osteomyelitis-incidence and differentiation from other acute onset musculoskeletal features in a population-based study. BMC Pediatr 2008;8:1-10. 18937840 10.1186/1471-2431-8-45 PMC2588573

[2] Rand N Mosheiff R Matan Y Porat S Shapiro M Liebergall M: Osteomyelitis of the pelvis. J Bone Joint Surg 1993;75:731-733. 10.1302/0301-620X.75B5.8376428 8376428

[3] ben Ghozlen H Kaziz H Abid F Zitoun Y Sassi N: Management of subacute acetabular osteomyelitis in a child. Arch de Pediatrie 2015;22:861-864. 10.1016/j.arcped.2015.05.001 26143999

[4] Kang SN Sanghera T Mangwani J Paterson JMH Ramachandran M: The management of septic arthritis in children: Systematic review of the English language literature. J Bone Joint Surg Ser B 2009;91:1127-1133. 10.1302/0301-620X.91B9.22530 19721035

[5] Castellazzi L Mantero M Esposito S: Update on the management of pediatric acute osteomyelitis and septic arthritis. Int J Mol Sci 2016;17:855. 10.3390/ijms17060855 PMC4926389 27258258

[6] Thomsen I Creech CB: Advances in the diagnosis and management of pediatric osteomyelitis. Curr Infect Dis Rep 2011;13:451-460. 21789499 10.1007/s11908-011-0202-z

[7] Peltola H Paä̈kk̈onen M: Acute osteomyelitis in children. New Engl J Med 2014;370:352-360. 24450893 10.1056/NEJMra1213956

[8] Scillia A Cox G Milman E Kaushik A Strongwater A: Primary osteomyelitis of the acetabulum resulting in septic arthritis of the hip and obturator internus abscess diagnosed as acute appendicitis. J Pediatr Surg 2010;45:1707-1710. 20713224 10.1016/j.jpedsurg.2010.04.013

[9] Howard-Jones AR Isaacs D: Systematic review of duration and choice of systemic antibiotic therapy for acute haematogenous bacterial osteomyelitis in children. J Paediatrics Child Health 2013;49:760-768. 10.1111/jpc.12251 23745943

[10] Chung WK Slater GL Bates EH: Treatment of septic arthritis of the hip by arthroscopic lavage. J Pediatr Orthopaedics 1993;13:444-446. 10.1097/01241398-199307000-00005 8370777

